# Theranostics application of tumor-initiating cell probe TiY in non-small cell lung cancer

**DOI:** 10.7150/thno.79282

**Published:** 2023-02-21

**Authors:** Yong-An Lee, Chee Chong Jonathan Lek, Gao Rong, Zhengwei Wu, S Shathishwaran, Jia Hui Jane Lee, Wai Leong Tam, Torsten Wuestefeld, Sung-Jin Park, Sangyong Jung, Beomsue Kim, Nam-Young Kang, Young-Tae Chang

**Affiliations:** 1 Genome Institute of Singapore (GIS), Agency for Science, Technology and Research (A*STAR), Singapore, 138672, Singapore.; 2 School of Biological Sciences, Nanyang Technological University, Singapore, 637551, Singapore.; 3 Cancer Science Institute of Singapore, National University of Singapore, Singapore, 117599, Singapore.; 4 Department of Biochemistry, Yong Loo Lin School of Medicine, National University of Singapore, Singapore, 117597, Singapore.; 5 National Cancer Centre Singapore, 169610, Singapore.; 6 Institute of Bioengineering and Bioimaging (IBB), Agency for Science, Technology and Research (A*STAR), Singapore, 138667, Singapore.; 7 Institute of Molecular and Cell Biology (IMCB), Agency for Science, Technology and Research (A*STAR), Singapore, 138673, Singapore.; 8 Neural Circuit Research Group, Korea Brain Research Institute (KBRI) Daegu, 41068, Republic of Korea.; 9 Department of Convergence IT Engineering, Pohang University of Science and Technology, Pohang, 37673, Republic of Korea.; 10 Center for Self-assembly and Complexity, Institute for Basic Science (IBS), Pohang, 37673, Republic of Korea.; 11 Department of Chemistry, Pohang University of Science and Technology (POSTECH), Pohang, 37673, Republic of Korea.

**Keywords:** Tumor-initiating cells, Non-small cell lung cancer, Fluorescent probe, cancer treatment

## Abstract

**Background:** Tumor-initiating cells (TIC) often elude conventional cancer treatment, which results in metastasis and cancer relapse. Recently, studies have begun to focus on the TIC population in tumors to provide better therapeutic options. Previously, we have reported the successful development of a TIC-specific probe TiY with the binding target as vimentin. While a low concentration of TiY showed a TIC visualization, at a high concentration, TiY induced selective toxicity onto TIC in vitro. In this study, we aim to assess TiY's applicability in theranostics purposes, from in vivo visualization to therapeutic effect toward TIC, in cancer mouse models.

**Methods**: We performed cell experiments with the TIC line model derived from resected primary non-small cell lung cancer (NSCLC) patient tumor. The animal model studies were conducted in mice of NSCLC patient-derived xenograft (PDX). TiY was intravenously delivered into the mice models at different concentrations to assess its in vivo TIC-selective staining and therapeutic effect.

**Results:** We demonstrated the TIC-selective identification and therapeutic effect of TiY in animal models. TiY treatment induced a significant ablation of the TIC population in the tumor, and further molecular study elucidated that the mechanism of TiY is through vimentin dynamics in TIC.

**Conclusion:** The results underscore the applicability of TiY for cancer treatment by selectively targeting soluble vimentin in TIC.

## Introduction

In recent years, tumor-initiating cells (TIC), more popularly known as cancer stem cells, have emerged as an essential target for eradicating cancer owing to their clinical implication of elevated resistance to conventional cancer therapy and metastasis 1. The epithelial-to-mesenchymal transition (EMT) is a widely studied biological program that confers mesenchymal traits on various types of epithelial cancer, including non-small cell lung cancer (NSCLC). The accumulated evidence has revealed that the EMT enables epithelial carcinoma cells to acquire the properties of TIC, such as increased metastasis and drug resistance upon mesenchymal trait acquisition 2. Therefore, developing strategies for identifying and therapeutically targeting mesenchymal-like carcinoma cells rather than their epithelial counterparts may produce a more durable anti-cancer response.

Fluorescent bioimaging probe development is a rapidly emerging field for identifying specific biological targets in various fields, including cancer research. We have developed a small molecule library composed of thousands of combinatorial fluorescent compounds and named Diversity-Oriented Fluorescent Library (DOFL). A distinct advantage of this library is its potential for use in high-throughput screening to identify and subsequently develop new bioimaging probes for various biological targets 3, 4. In previous work, the successful application of DOFL screening has been validated in cancer research by demonstrating TIC-specific probe development, TiY, through unbiased cell-based DOFL screening using reliable models of lung TIC 5. The extensive validations demonstrated the superior selectivity of TiY toward TIC via binding to vimentin, which has been acknowledged as a universal biomarker of EMT 6. Interestingly, at an increased concentration, TiY also showed selective cytotoxicity to the TIC over non-TIC cancer and normal epithelial cells, suggesting its validity in theranostics application through the dual potential in TIC selective visualization and therapeutic effect. Nevertheless, the in vivo applicability of TiY has yet been addressed.

In this study, we demonstrated the utility of TiY for targeting TIC in vivo. With the aggressive lung cancer models, we assessed TiY for TIC selective staining and the therapeutic effect in the mouse models. Repeated treatment of TiY inhibited tumor formation by targeting vimentin-positive cells in tumors with low toxicity to normal cells. We finally elucidated the molecular mechanism of selective toxicity to TIC in terms of vimentin dynamics. By analyzing the dynamic status of vimentin between soluble and insoluble forms, we revealed an increased proportion of soluble vimentin in TIC compared to normal cells. These provided the molecular level mechanism for the TIC-selective toxicity over the normal mesenchymal tissues.

## Materials and methods

Details of the chemical, including the synthesis procedure, were described in the previous report 5. All mouse experiments in the present study were conducted with approval by the Agency for Science, Technology, and Research of Singapore-Biological Resource Centre IACUC (protocol number 211598). The datasets used during the current study are available from the corresponding author on reasonable request.

### Ethics approval and consent to participate

All human tumor tissue samples were collected in accordance with national and institutional ethical guidelines.

### Cell cultures

Unless otherwise stated, all reagents for cell cultures were purchased from Thermo Fisher Scientific. Tumor sphere culture was maintained in serum-free condition as described previously 5. Human dermal fibroblast cells (Lonza) were maintained in DMEM containing 10% FBS and 2 mM L-glutamine 1% antibiotics. The fibroblast cell line was passaged by trypsin-EDTA digestion, and the fresh medium was replenished every 3 days.

### Flow cytometry and fluorescence-activated cell sorting

Cells were incubated with antibodies against CD166-Cy5 (Thermo Fisher Scientific) or TiY at the concentration of 1 μg / mL and 10 nM in 1 mL of DPBS containing 2% FBS and antibiotics (DPBS-S) containing 1 × 10^6^ cells for more than 40 min at 4°C. The cells were washed and resuspended in the DPBS-S. The fluorescence intensity was measured on flow cytometry (BDTM LSR II), or FACS was performed on a FACSAria II (BD) for cell sorting.

### Tumor sphere formation assay

FACS-purified cells were seeded in non-culture-treated 12 well plates. The cells were maintained in the plates containing TS culture media at 37 ℃, 5% CO2, and the number of spheres was counted under the microscope after 4-7 days. Spheres were counted if the size is larger than 50 μm.

### TS forming inhibition test of TiY

For in vitro test of TiY on TS forming inhibition, spheres of TS32 were enzymatically dissociated and seeded in different wells of the TS culture plate and cultured with TiY at different concentrations. Number of spheres was counted after culture for 7 days.

### Ex vivo TIC imaging with TiY in lung cancer mouse model

To generate the lung cancer mouse model, TS32 cells or lung cancer patient-derived xenografts (PDX) line A139 cells (5 × 10^5^) were injected into the tail vein of NOD.Cg-PrkdcscidIl2rgtm1Wjl/SzJ (NSG) mice. TiY was dissolved in 100% DMSO and was further diluted at 1:1000 in the vehicle solution (PBS, 0.01% BSA, 0.02% Tween-20). At the 4-6 weeks following injection, TiY was administrated to normal or the lung cancer mouse models (10 μL / g) by tail vein injection two hours before imaging. The mice were euthanized to scan lungs for the fluorescent ex vivo. The fluorescent was measured by In Vivo Extreme II with a wavelength of ex540-em600 (Bruker). The fluorescence intensity was calculated by molecular imaging software MI (Bruker). The tumor formation in the lung was confirmed by subjecting the cells harvested from single-cell dissociation of the lung lobes for the tumor sphere-forming assay. The cells were washed and resuspended in the DPBS containing 1% FBS. TiY-dependent sorting was performed on a FACSAria II (BD) to assess the difference in the tumor sphere-forming ability between TiY bright (+) and dim (-) cells. To confirm the tumorigenicity of the cultured spheres, the spheres were injected into the mouse subcutaneously after single-cell dissociation.

### Therapeutic effect study of TiY

To study the therapeutic effect of TiY for targeting TIC, 5 × 10^5^ cells of the PDX-A139 line were co-injected with Geltrex (Thermo Fisher Scientific) into the flank site of NSG mice. A day after injection, 100 or 300 μM of TiY (10 μL / g) was administrated by tail vein injection, and the tumor size was recorded every three days until day 28. The control group was given the same volume of vehicle solution. On day 31, the tumors were harvested from the mice and weighed.

### Further analysis of tumor cells harvested from TiY-treated PDX mice

To assess the difference in tumorigenicity between tumors of the two groups, TiY treated and the control mice, single cells were dissociated from each tumor and subjected to the tumor sphere-forming and the secondary transplantation assay. For the secondary transplantation assay, 5 × 10^5^ tumor cells were prepared with Geltrex after being harvested from two groups and followed by subcutaneous injection into new NSG recipient mice. The mice were observed for tumor growth to compare the tumorigenicity of the tumor cells between TiY-treated and control groups.

### Toxicity test of TiY

Cell suspensions were inoculated in 96-well plates and treated with different concentrations of TiY. After 72 hr cell maintanence at 37 ℃, 5% CO2, cells were incubated with CCK solution (Dojindo) per well for 2 hr. Absorbance was determined at 450 nm using a fluorescence microplate reader (Promega™ GloMax®). The percentage of cell proliferation was calculated by converting the absorbance to percentage of control.

### Analysis of soluble and insoluble vimentin in TS32 and normal mesenchymal tissue cells

To separate the soluble and insoluble fractions from the cells, TS32 and HDFCs were incubated with a lysis buffer containing a proteinase inhibitor cocktail, respectively. After centrifugation at 17,800 g for 10 minutes at 4°C, the supernatant (soluble fraction) was collected. The precipitates (insoluble fraction) were resuspended in an SDS-lysis buffer and sonicated. Both fractions were reconstituted to equal volumes, and 15 uL of each fraction was subjected to western blot analysis. Proteins were separated by SDS-PAGE, transblotting, and subsequent immunoreactions using chemiluminescence. The antibody for vimentin (1:3000, Cat# 50-9897-82, Thermo Fisher Scientific) was used in this study. GAPDH was used as a loading control.

### Fluorescence scan of SDS-PAGE gel

Cell pellet of TiY-treated TS32 and 32A were lysed with a lysis buffer (Thermo Fisher Scientific) premixed with 10 μL/mL Protease Inhibitor Cocktail (GE healthcare). The supernatant was collected by centrifuge at 16,000 × g for 20 min at 4 °C and protein concentration was determined by Bradford protein assay reagent (Bio-Rad). Each sample was mixed with 4× Laemmli sample buffer (Bio-Rad) and analyzed by SDS-PAGE after boiling at 95°C for 5 min. To find the fluorescence bands for TiY, in gel fluorescence was scanned with a Typhoon (GE healthcare).

### RNA Sequencing

The RNA was extracted from TS32 and 32A cells using the RNA extraction kit, RNeasy kit (Qiagen), according to the manufacturer's instructions. cDNA libraries were prepared from total RNA using the TruSeq stranded mRNA library kit (Illumina) according to the manufacturer's instructions. RNA sequencing was performed by NextSeq High (Illumina) using one lane of a 1 × 76 bp (multiplexed) run. The data were generated after mapping, and subsequently FPKM values were calculated by Cuffdiff. The Heatmap of RNA sequencing data was generated using MORPHEUS (https://software.broadinstitute.org/morpheus/).

### Statistical Analysis

All experimental results are presented as means ± SEM, if not stated otherwise in the figure legend. Statistical significance was calculated using Excel (Microsoft) and GraphPad Prism 9. It was evaluated by the Student's t-test and considered to be statistically significant when p < 0.05 as denoted by an asterisk in the graphs.

## Results and Discussion

### Dual potential of TiY in TIC-selective visualization and therapeutic effect

**Figure 1:**
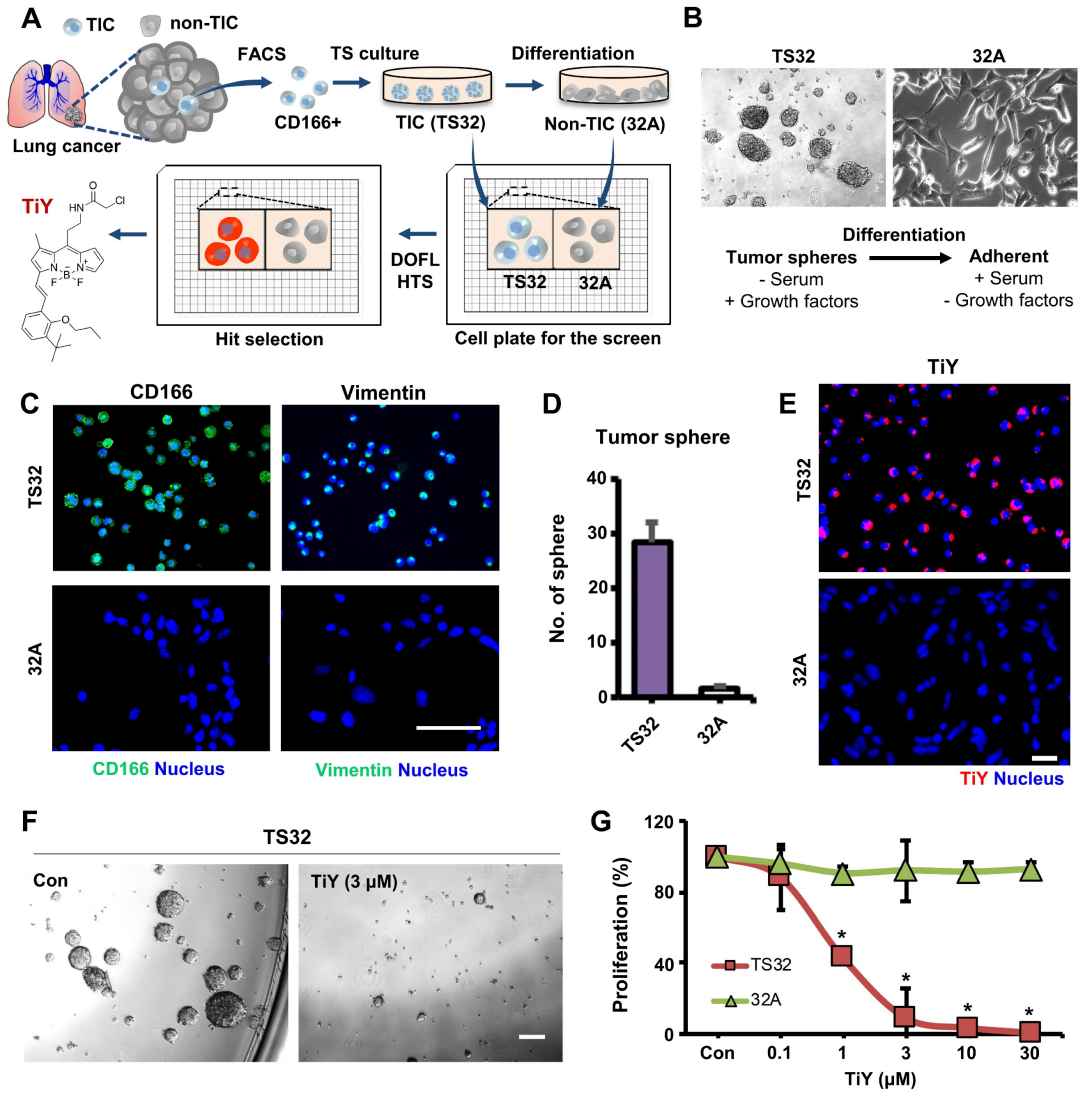
**Theranostic applications of TiY with the dual potential of TIC-selective visualization and therapeutic effect. (A)** Schematic process of TIC-specific probe development. TiY was developed as a TIC-specific probe through DOFL screening with cell models of lung TIC and non-TIC cancer cells. TS32 cell line is established as a TIC line by using antibodies against to lung TIC marker CD166 from tumor cells of the lung cancer patient. An isogenic counterpart of the TS32 cells, 32A, was derived as a non-TIC cancer tumor cell model by inducing differentiation in the media which removed growth factors from TIC culture media. **(B)** Two cell lines were used as TIC and non-TIC tumor cell line models, respectively. TIC model line, TS32 (left), was maintained as tumor spheres in a serum-free medium containing growth factors. Non-TIC tumor cell line model was derived from tumor spheres of TS32 as the differentiated cells (32A: right) that were generated through a repetitive passage of TS32 in a serum-supplemented medium without the growth factors. **(C)** Fluorescence images of immunofluorescent staining against CD166 and vimentin in TS32 and 32A cells, respectively. TS32 is a highly enriched cell line for the CD166^+^ and vimentin^+^ cells, while 32A cells show loss of CD166 and vimentin expression after differentiation. Scale bar, 50 μm. **(D)** Comparison of sphere-forming ability between TS32 and 32A cells in tumor sphere culture condition. Values are means ± SEM (n=3). **(E)** TiY selective stains TS32 cells over its isogenic counterpart control 32A cells. **(F)** TS cell culture with TiY. TiY inhibited sphere formation at increased concentrations. Scale bar, 250 μm. **(G)** Anti-TIC activity of TiY over non-TIC. TiY showed a TIC-selective inhibition effect towards TS 32 cells over 32A cells at higher concentrations, while low concentrations did not.

TiY was developed as a lung TIC probe through DOFL screening with reliable cell models of lung TIC and non-TIC tumor cell lines. The Lung TIC line was established by fluorescence-activated cell sorting (FACS) of the TIC population with CD166 antibody, which has previously shown robustness as a lung TIC marker 7, 8, from resected primary NSCLC adenocarcinoma patient tumors. The CD166^+^ cells were grown as tumor spheres in our culture condition with a serum-free medium containing growth factors and maintained as a lung TIC line, TS32. As the corresponding isogenic control to TS32, adherent type cells were generated as a non-TIC tumor cell line, 32A, by differentiation from TS32 cells through a repetitive passage in serum-containing media withdrawn the growth factors (Figure 1A, B). The TS32 is a highly enriched line for TIC, as demonstrated that the cells harvested from TS32 (TS32 cells) retained in vitro sphere-forming ability, which is a standard in vitro surrogate assay to assess the self-renewal capacity of NSCLC 8, 9, and a high expression level of CD166. As a distinct population in the adenocarcinoma, which refers to epithelial origin, TIC often retains mesenchymal traits and shows EMT biomarker expression, including vimentin. It was known that the vimentin level in tumor cells correlates well with tumor aggressiveness and poor prognosis 6, 10. Our immunofluorescence observation in TS32 cells showed vimentin expression, thus demonstrating that the TS32 cells are an EMT-derived population. In contrast, 32A cells were barely capable of sphere-forming as well as loss of CD166 and vimentin expression (Figure 1C, D). Several biomarkers have been identified over the last decades that can discriminate TIC from non-TIC populations in tumors. Stemness-related genes and EMT markers have been validated as TIC markers due to their positive correlations with tumorigenicity and metastasis 2, 10-12. We performed RNA sequencing of TS32 and 32A cells and compared their TIC marker gene expressions. The result showed increased expression of stemness-associated genes and EMT markers in TS32 compared to 32A cells (Figure S1). This result additionally supports the reliability of our cell models as TIC and non-TIC tumor cell line.

The function of TiY in TIC-selective visualization was demonstrated by showing clearly that TiY stains TS32 cells much brighter over its counterpart 32A cells (Figure 1E). We previously suggested that TIC-selective staining of TiY over non-TIC cancer cells was facilitated via binding to vimentin 5. To validate the vimentin specificity of TiY, the lysates of TS32 and 32A cells were extracted after incubation with TiY and subjected to SDS-PAGE for a fluorescence scan of TiY. Among the whole proteome, we found only a single strong band at the vimentin protein size, which is about 54 kDa, in TS32 cell lysate, whereas no band was observed in the 32A cell lysate (Figure S2A). We have further confirmed the diminished TiY signal in the TS32 cells after the knock-down of vimentin with siRNA (TS32-VIM in Figure S2B), suggesting that vimentin is the sole responsible binding target of TiY in TIC cells.

We speculated the entry mechanism of TiY would be either active transporter dependent, which requires cellular energy, or passive manners 13. To check the possibility, we compared the cell uptake of TiY in live cells and PFA (paraformaldehyde)-fixed cells (dead cells, but with intact membrane structure). Notably, TiY could stain both alive and fixed TS32 cells, maintaining the selectivity over 32A cells (Figure S3A). Thus we concluded that TiY goes into the cells passively, which does not require energy to transport molecules across cell membranes. To understand how long the staining takes, we measured the kinetics of the fluorescence intensity of the cells upon spike of TiY (Figure S3B). The result showed that the intensity of TiY quickly reached the maximum level within 20 min.

One other function that TiY possess is the TIC-selective therapeutic effect. When we grew TS32 cells with TiY at increased concentration, sphere-forming was dramatically repressed (Figure 1F). Interestingly, this inhibitory effect was TIC-selective over non-TIC cells, which was demonstrated by showing the result that TS32 cells were susceptible to the cytotoxicity of TiY much more than 32A cells (Figure 1G). We noted the observation that the therapeutic effect was observed from the high concentrations with an apparent inhibitory effect from 3 μM, while low concentrations of 0.1 μM did not show. Thereby, it is suggested that TiY is a fluorescent compound for theranostics application with dual potential in TIC-selective visualization and therapeutic effect by applying different concentrations in lung cancer, depending on its use.

### In vivo study of the TiY selectivity towards TIC in lung cancer mouse model

To determine whether TiY facilitates TIC enrichment from diverse samples, including NSCLC adenocarcinoma patient-derived xenograft (PDX) line A139 5, we harvested tumor cells from A139-derived tumor-bearing mice to apply fluorescence-activated cell sorting (FACS). We first checked the correlation of TiY staining and vimentin expression level from PDX A139 cells and demonstrated that they are proportionally correlated in a positive manner from flow cytometry analysis (Figure S4A). We subsequently performed FACS to sort the TiY^+^ and TiY^-^ populations from PDX A139 cells (Figure S4B) and subjected the sorted cells to immunofluorescence staining for vimentin. The result clearly showed a successful enrichment of vimentin-expressing cells in the TiY^+^ over TiY^-^ (Figure S4C). The TiY^+^ cells were generally more tumorigenic, and the TIC characters were validated in the tumor sphere formation assay (Figure S4D and E). Notably, when compared to the lung TIC marker CD166, TiY showed a significantly greater propensity for enriching the TIC population in the PDX A139 cells, thereby demonstrating the superiority of TiY in TIC recognition to anti-CD166. The greater tumorigenicity of the TiY^+^ cells was also validated by transplantation into the recipient NSG mice (Figure S4F). We performed the immunofluorescence analysis for vimentin expression in the TiY^+^ and TiY^-^ cells-derived tumors and observed highly enriched vimentin-expressing cells in the TiY^+^-derived tumors than in the TiY^-^-derived tumor (Figure S4G).

**Figure 2:**
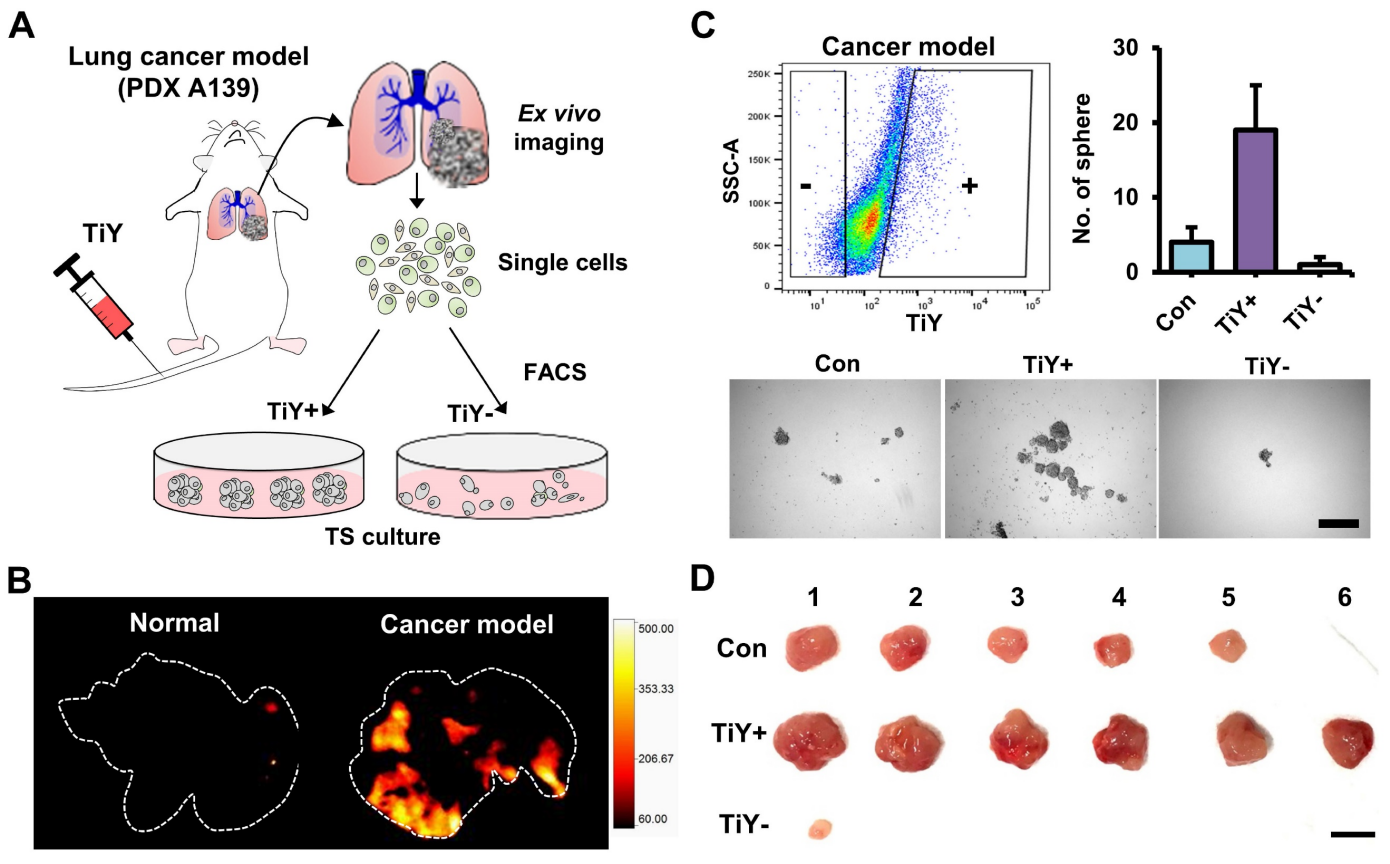
**Assessment of TiY for selectivity towards TICs in vivo. (A)** Schematic diagram of the experimental procedure for validating TiY's selectivity for TIC in lung cancer model mice. The model was generated by tail vein injections of lung cancer PDX line A139 cells. Following 4-6 weeks of post-implantation, 100 μM TiY was administrated by tail vein injection. One hour after the injection, the lung was harvested to follow ex vivo fluorescence imaging for TiY and TiY dependent sorting to assess the tumor sphere-forming ability after single-cell dissociation, respectively.** (B)** Ex vivo fluorescent image of the lung for TiY. **(C)** The result of a tumor sphere-forming assay of each fraction (TiY^-^ and TiY^+^) in lung lobe cells dissociated from the lung of the cancer model. Values are means ± SEM (n=2). Scale bar, 500 μm. **(D)** The tumorigenicity assessment of the tumor spheres harvested from (C). Scale bar, 1 cm.

Furthermore, to evaluate the TIC-selectivity in vivo, we first generated a mouse model by tail vein injections of A139 cells into NSG mice. Following 4-6 weeks of post-implantation, TiY was intravenously delivered to the cancer model and normal mice, followed by lung harvest from the mice for ex vivo fluorescent imaging of lung tissues (Figure 2A). As expected, TiY fluorescence was significant in the lungs of the cancer model compared to the normal mice (Figure 2B). After single-cell dissociation of the lung lobes, cells were sorted into TiY^+^ and TiY subpopulations and subjected to a tumor sphere-forming assay. The TiY^+^ cells displayed a strong tumor sphere-forming ability compared to the TiY^-^ cells (Figure 2C). As TIC is functionally defined by its ability to form new tumors by seeding a small number of cells in the appropriate recipients, the tumor spheres were further validated for tumorigenicity by sequential transplantation into other NSG mice (Figure 2D). The result clearly showed that tumor formation in the TiY^+^ fraction injected mice, demonstrating that TiY has profound selectivity towards the TIC population in vivo.

### Applicability of TiY for TIC targeting in vivo

To evaluate TiY for TIC targeting therapeutic effect in vivo, we preliminarily tested a concentration of 100 μM in the TS32 cells-derived tumor mice. We implanted TS32 cells into the flank site of NSG mice to generate the tumor mouse models. TiY and the vehicle without TiY (control) were intravenously administered from the following day of the TS32 cell implantation in 3-day intervals for 28 days (Figure S5A). Although there was a decreased tumor sphere (TS) forming capacity in the TiY-treated tumors in the later analysis, no initial visible effect on the tumor size was observed (Figure S5B and C). Based on the negative observation, we decided not to test TiY at lower concentrations than 100 μM and tried to adopt practically maximal concentration (due to the hydrophobicity) for the formulation of injection, which was 300 μM.

**Figure 3:**
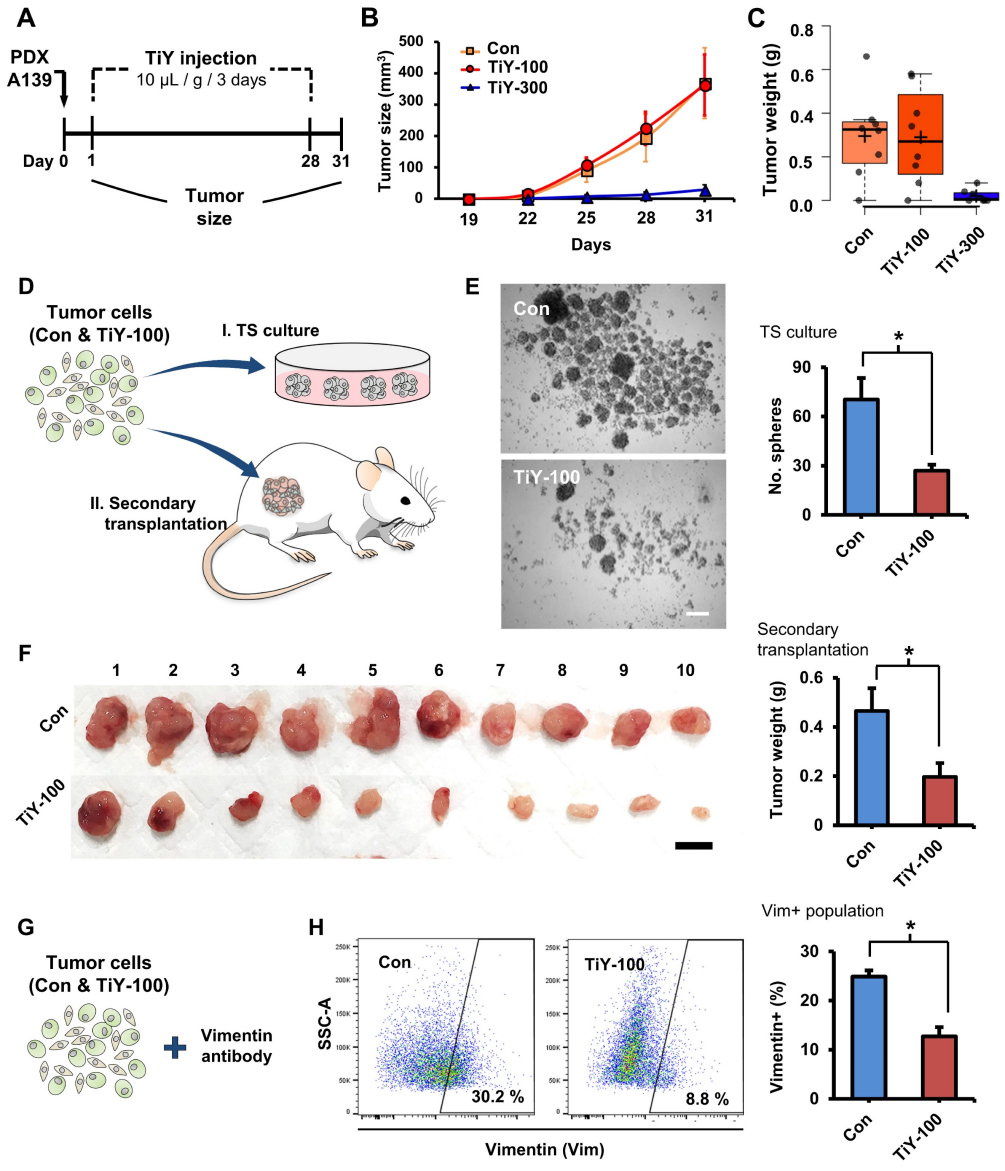
** The therapeutic effect of TiY for targeting TIC in vivo. (A)** Schematic diagram of the experimental procedure for validating the therapeutic effect of TiY against TIC in lung cancer PDX mice. One day after the subcutaneous transplantation of A139 cells into the mice flank, 100 and 300 μM of TiY (TiY-100 and -300) were administrated by tail vein injection every three days (10 μL/ gram body weight) until day 28. The tumor size was recorded until day 31, after which tumors were harvested for analysis. The same volume of the vehicle solution was given to the control group mice without TiY (Con). **(B-C)** The size and weight of tumors harvested from con-, TiY-100, and -300 groups. Values are means ± SEM (n=8). **(D)** A schematic diagram of the experimental procedure for the additional analysis of tumors from the con- and TiY-100 groups. To compare the TIC frequency between the two groups, the tumor sphere-forming assay, and the secondary transplantation assay were performed with tumor cells harvested from the two groups. **(E)** The result of the tumor sphere-forming ability and the secondary transplantation assays of the Con- and TiY-100 groups tumor. Values are means ± SEM (n=10). Scale bar, 250 μm. **(F)** The result of the secondary transplantation assay of the tumor cells harvested from con- and TiY-100 groups, respectively. Scale bar, 1 cm. **(G, H)** Flow cytometry analysis for vimentin expressing cell population in TiY-100 tumors compared to Con tumors. Values are means ± SEM (n=10).

As a subsequent therapeutic study, we used PDX tumor mice, which have been known to reflect the true behavior of the patient tumor more than cell line xenografts. Based on the result of the preliminary study with TS32 line-derived tumor mice, we examined the possibility of inhibiting tumor initiation with an increased concentration of TiY 300 uM in PDX A139 tumor mice. We administered 100 and 300 uM TiY (TiY-100 and 300, respectively) and the vehicle without TiY (con) intravenously (Figure 3A). As expected, the higher dose, TiY-300, successfully inhibited tumor formation in the mice (Figure 3B and C). Whereas, as expected, no significant size reduction was observed in TiY-100 mice compared to the control mice. However, based on our preliminary observations; I. when the cells were exposed to TiY in short-term less than 24 hours, the high concentrations of TiY (3 uM and above) significantly increased the apoptotic population and successfully inhibited sphere formation in the tumor sphere culture assay, while the lower concentrations of TiY (1 uM and below) showed no cytotoxic effect; II. the therapeutic effect of TiY had been increased by the extension of exposure time of the same concentration of TiY (Figure S6A and B); III. When the gene expression levels were investigated by qRT-PCR in the TiY-treated TS32 cells, downregulation of the critical TIC-associated genes, Nanog, Snail, and N-Cadherin, was observed in response to TiY treatment (Figure S6C), we speculated that the repetitive injections of the lower dose might yet alter the TIC characteristics. To demonstrate this, we harvested cells from the TiY-100 and control tumors for further analysis in tumorigenicity, including tumor sphere-forming assay and the transplantation of the tumor cells into new recipient NSG mice (secondary transplantation assay) (Figure 3D). Interestingly, TiY-100 group tumors showed a significantly decreased tumor sphere-forming capability compared to the control (Figure 3E). Upon secondary transplantation of the TiY-100 group tumor cells, there was a remarkable reduction in tumor growth in comparison to the control (Figure 3F). It is noteworthy that flow cytometric analysis of the tumor cells showed a decreased population of vimentin-positive cells in the TiY-100 tumors (Figure 3G and H). As TiY is a vimentin-binding probe, it was no surprise to observe a decrease in TiY^+^ populations in the TiY-100 tumors compared to the control (Figure S7A). The decrease in the TiY^+^ populations could be attributed to the decrease in tumorigenicity of these TIC. This notion is supported by previous studies which demonstrated the benefits of targeting the mesenchymal TIC in epithelial carcinoma tumors 14. The deceased frequency of TIC in the TiY-100 was additionally validated by measuring a positive cell population against the lung TIC marker protein CD166 (Figure S7B). The data showed that 28% of CD166^+^ cells are in the control tumor cells, and a significant decrease was observed in the TiY-100 tumor cells (11%).

### Toxicity assessment of TiY on normal mesenchymal tissue cells

**Figure 4:**
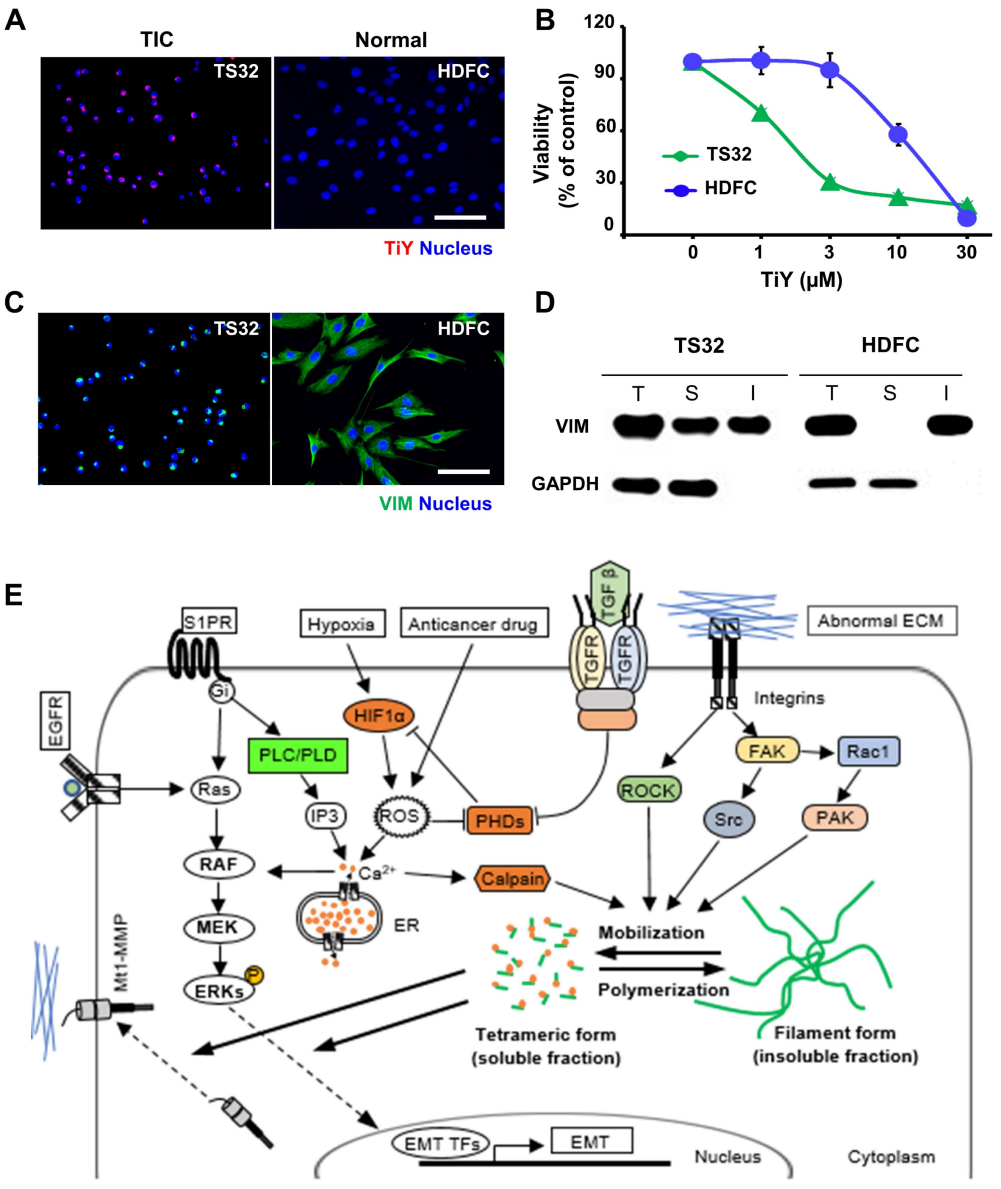
** Selectivity of TiY toward TIC over normal mesenchymal tissue cells. (A)** Fluorescence images of TS32 and normal tissue cells, HDFC, with TiY. Scale bar, 100 μm. **(B)** Comparison of cytotoxicity of TiY on TS32 cells and HDFC. Values are means ± SEM (n=3). **(C)** Immunofluorescence for vimentin expression in TS32 cells and HDFC. Scale bar, 100 μm. **(D)** Western blotting analysis of vimentin soluble (S) and insoluble (I) protein fractions of TS32 and HDFC. The T refers to the total. GAPDH is a soluble protein. **(E)** The role of soluble vimentin in cell phenotypic regulation. Soluble vimentin, as the mobilized form of vimentin, has been found to regulate cellular molecules, including Mt1-MMP and ERKs, that were found to regulate EMT in different types of cancer. It was demonstrated that the structural dynamics from the filament form (insoluble fraction) to the tetrameric form (soluble fraction) could be induced by diverse cellular molecules, including calpain and kinases, which are found to promote malignancy in different types of cancer.

To circumvent the possibility that TiY may affect the activity of normal cells, we performed the toxicity test on the normal mesenchymal tissue cells. The normal human dermal fibroblast cells (HDFC) and TS32 were grown respectively, and we first confirmed by fluorescent imaging that TiY stains TS32 selectively over the normal cells (Figure 4A). We thereafter compared the cell growth with different concentrations of TiY for the toxicity test. The result clearly showed that the HDFC substantially tolerated higher doses of TiY without a prominent toxic feature in contrast to the remarkable inhibition in the TS32 cells (Figure 4B). However, when checking the vimentin contents in the two cells, the HDFC cells expressed an even higher level of vimentin than the TS32 cells, which was opposite to our expectation (Figure 4C). To address the puzzle, we further analyzed the vimentin status in both cells.

Basically, two forms of vimentin exist in cells, soluble tetrameric and insoluble filaments form 15, 16, and we found that the soluble fraction was significantly increased in TS32. In contrast, vimentin was mainly in an insoluble form in HDFC (Figure 4D). We previously suggested that TiY binds to the tetrameric form of vimentin 5. In this study, to validate the specific binding of TiY to the soluble vimentin over its insoluble counterpart, we stained TS32 and HDFC cells with TiY and separately isolated soluble and insoluble proteins (Figure S8A). The soluble fraction of TS32 clearly showed a much higher fluorescence over all other fractions (Figure S8B), demonstrating the specific binding of TiY onto the soluble form. In addition, we checked the total amount of vimentin in soluble and insoluble form from TiY-stained TS32 cell populations. While TS32 cells are mostly vimentin^+^, there were clearly two populations of TiY^+^ and TiY^-^ (Figure S8C). The two populations were isolated (Figure S8D) from the TS32 cells and subjected to further analysis for the vimentin fractions. Interestingly, the TiY^+^ population has a much higher fraction of soluble vimentin compared to that of TiY^-^ cells (Figure S8E). As expected, the result of the tumor sphere assay showed that TiY^+^ cells are clearly more tumorigenic than the TiY^-^ population in TS32 cells (Figure S8F). Taken together, these results demonstrate that selective-staining and -toxicity of TiY onto TIC is through the increased level of the soluble form of vimentin.

Although the architectural role has long been considered a central function of vimentin, recent studies showed that the cellular network of vimentin is also critical in cellular phenotypic regulation 17. The structural dynamics of vimentin between the filament and tetramer form have been found to facilitate intracellular communication 18. The direct interplays of soluble vimentin with signaling molecules, such as membrane type 1-matrix metalloproteinase (Mt1-MMP) and extracellular signal-regulated kinases (ERKs), are likely to enhance tumorigenicity in different types of cancer 19. Although the exact functional role of the soluble vimentin in biological events in the TIC has not yet been fully understood, it was demonstrated that the signaling pathways responsible for the vimentin mobilization from the filament form (insoluble fraction) into the tetrameric form (soluble fraction) were heightened predominantly in malignant cancers (Figure 4E) 19-25.

## Conclusion

Our results demonstrate the capacity of TiY as a chemical probe for TIC-selective visualization in lung cancer model mice at low concentrations as well as a pharmacological agent for treating tumors at high concentrations. In vivo treatment of TiY exhibits a therapeutic effect on lung cancer tumors via targeting TIC expressing soluble vimentin. While repetitive administration of the lower concentration (100 μM) declined TIC activity of the tumor cells, high dose TiY treatment (300 μM) directly inhibited tumor formation. The mechanism study explored in this study showed the increased proportion of soluble vimentin in TIC compared to normal tissue cells, which elucidates the reason for the selective therapeutic effect of TiY on TIC over normal cells expressing vimentin. This study reveals a new direction for novel drug development targeting the soluble vimentin for efficient cancer treatment.

## Supplementary Material

Supplementary figures.
