# Supplementary material for: Theranostics application of tumor-initiating cell probe TiY in non-small cell lung cancer
Source: Theranostics. 2023 Feb 21;13(4):1370–80. doi: 10.7150/thno.79282 (PMC10008731; doi:10.7150/thno.79282)
Supplement: Supplementary file 1 — Supplementary figures. [file thnov13p1370s1.pdf]

Supplementary material for

Theranostics application of tumor-initiating cell probe TiY in non-small cell lung cancer

Running title: Fluorescent chemical probe targeting tumor-initiating cells

Yong-An Lee[1], Chee Chong Jonathan Lek[1,2], Gao Rong[1], Zhengwei Wu[1,3], S Shathishwaran[1], Jia Hui Jane Lee[1,2], Wai Leong Tam[1,2,3,4], Torsten Wuestefeld[1,2,5], Sung-Jin Park[6], Sangyong Jung[7], Beomsue Kim[8], Nam-Young Kang[9*], Young-Tae Chang[10,11*]*

[1]Genome Institute of Singapore (GIS), Agency for Science, Technology and Research (A*STAR), Singapore, 138672, Singapore.

[2]School of Biological Sciences, Nanyang Technological University, Singapore, 637551, Singapore.

[3]Cancer Science Institute of Singapore, National University of Singapore, Singapore, 117599, Singapore.

[4]Department of Biochemistry, Yong Loo Lin School of Medicine, National University of Singapore, Singapore, 117597, Singapore.

[5]National Cancer Centre Singapore, 169610, Singapore

[6]Institute of Bioengineering and Bioimaging (IBB), Agency for Science, Technology and Research (A*STAR), Singapore, 138667, Singapore.

[7]Institute of Molecular and Cell Biology (IMCB), Agency for Science, Technology and Research (A*STAR), Singapore, 138673, Singapore.

[8]Neural Circuit Research Group, Korea Brain Research Institute (KBRI) Daegu, 41068, Republic of Korea.

[9]Department of Convergence IT Engineering, Pohang University of Science and Technology, Pohang, 37673, Republic of Korea.

[10]Center for Self-assembly and Complexity, Institute for Basic Science (IBS), Pohang, 37673, Republic of Korea

[11]Department of Chemistry, Pohang University of Science and Technology (POSTECH), Pohang, 37673, Republic of Korea.

*Correspondence: **Young-Tae Chang***

Department of Chemistry, Pohang University of Science and Technology (POSTECH), Pohang, 37673, Republic of Korea.

E-mail: ytchang@postech.ac.kr.

Supplementary Data

Figure S1. RNA sequencing analysis of the TIC and non-TIC models

Figure S2. Binding target assessment of TiY as vimentin in the TIC

Figure S3. The staining mechanism of TiY

Figure S4. Selectivity assessment of TiY toward the TIC population in the lung cancer PDX line cells

Figure S5. Preliminary study of the TIC targeting effect of TiY in TS32 xenograft mice

Figure S6. TIC inhibition effect of TiY in TS32 cell culture with different concentrations and treatment hours

Figure S7. Flow cytometry analysis of tumor cells harvested from control and TiY-100 group

Figure S8. The selective binding of TiY onto the soluble vimentin

Supplementary Figure 1.

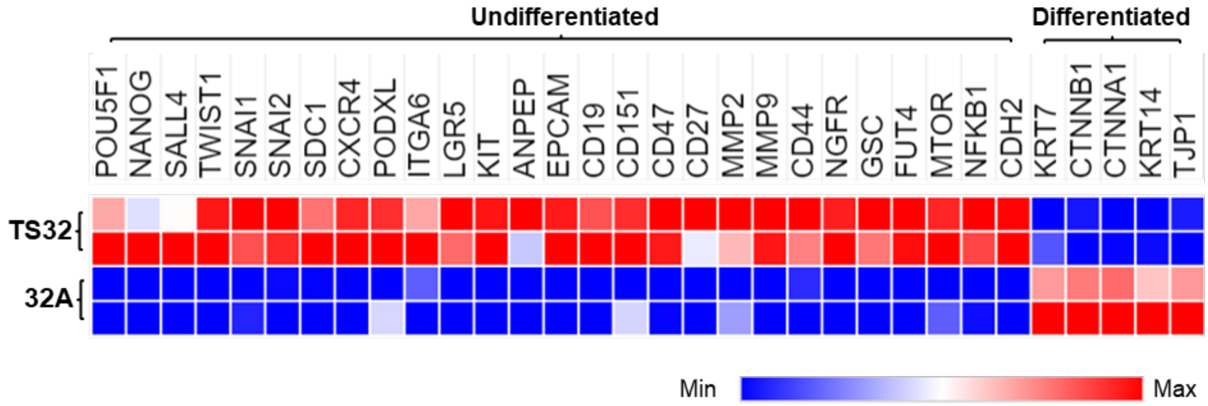

Figure S1. RNA sequencing analysis of the TIC and non-TIC models

The expression level of the selected TIC-related marker genes was depicted as a heat map based on the fragment per kilobase million (FPKM) value of the RNA sequencing analysis of the TIC (TS32) and non-TIC (32A) models. The TIC-associated (undifferentiated) and non-TIC-associated (differentiated) marker genes were selected from previous reports [2, 10-12]. The data showed that markers for stemness-associated genes, such as Oct4 (POU5F1) and Nanog, and EMT markers, were up-regulated in TS32 cells, while the differentiation markers were down-regulated compared to the 32A cells.

Supplementary Figure 2.

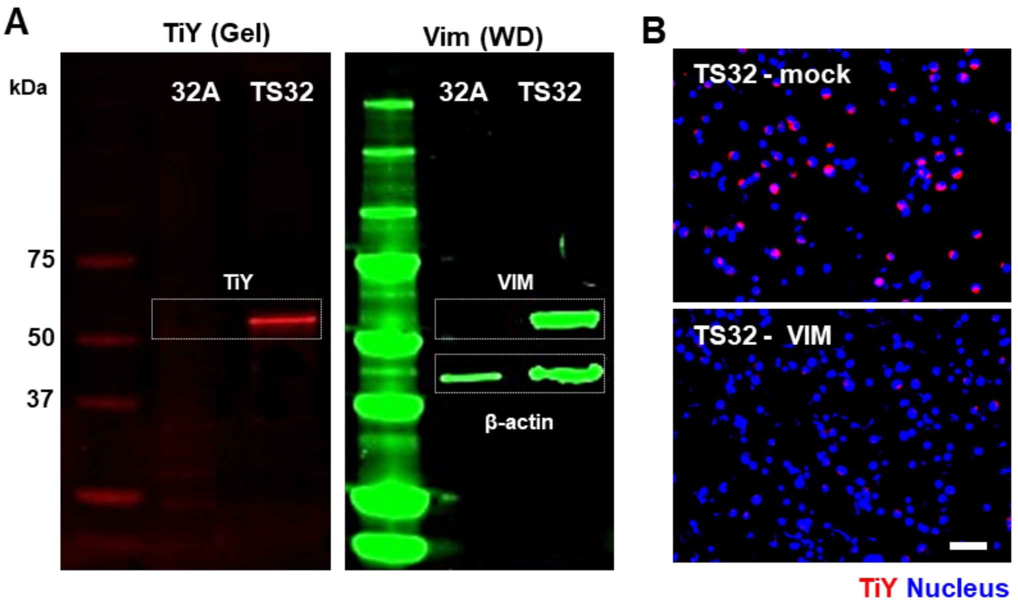

Figure S2. Binding target assessment of TiY as vimentin in the TIC and non-TIC cancer model

(**A**) Fluorescence scan of gel (left) subjected for 1D SDS-PAGE run with lysate extracted from TiY prestained 32A and TS32, respectively, and Western blotting result (right) showing vimentin (VIM) expression in the TS32 lysate. After the scan for the fluorescence bands detection for TiY in the SDS-PAGE gel, western blotting was performed with the gel to confirm vimentin bands in 32A and TS32 protein samples. The β-Actin antibody was used to confirm that the two protein samples were loaded accordingly. The antibody staining for β-Actin was started on the same membrane after scanning the vimentin protein band. (**B**) Fluorescence microscopic observation of TiY signal in TS32 cells after transfection with empty vector (TS32 - mock) and siRNA for vimentin (TS32-VIM). Scale bar, 50 μm.

Supplementary Figure 3.

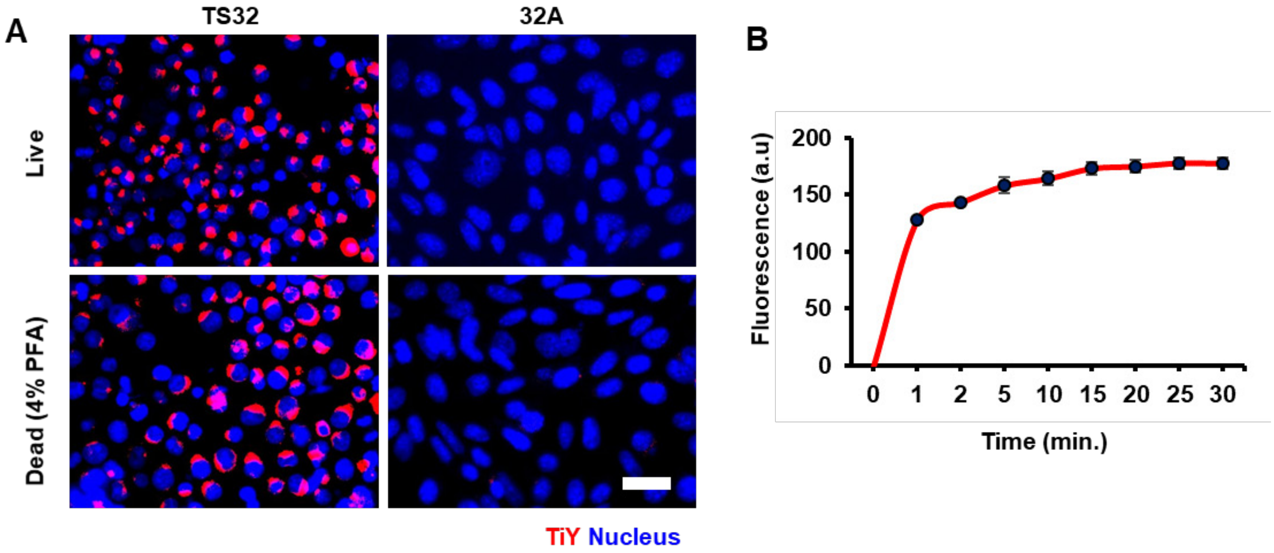

Figure S3. The staining mechanism of TiY

(A) TIC-selective staining of TiY in both live (Live; top) and dead by 4% PFA treatment (Dead). Scale bar, 50 μm. (B) Kinetic measuring of the fluorescence intensity of the cells upon spike of TiY. Values are means ± SEM (n=3).

Supplementary Figure 4.

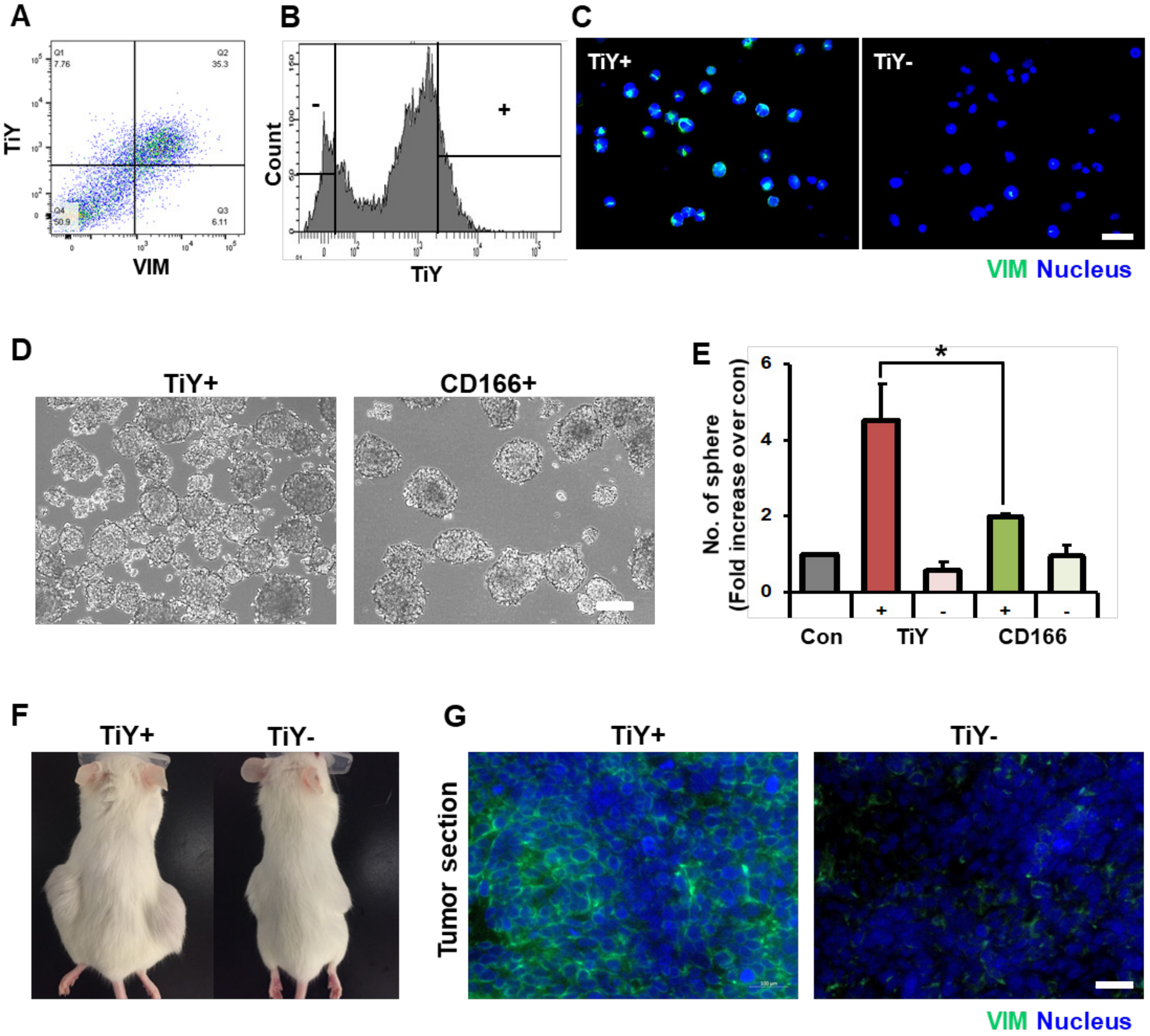

Figure S4. Selectivity assessment of TiY toward the TIC population in the lung cancer PDX line cells

(**A**) Dual staining of TiY and vimentin antibody in lung cancer PDX line A139 cells showing a positive correlation between vimentin expression and TiY$^+$ cell population. (**B**) The PDX A139 cells were subjected to TiY-dependent sorting using FACS after staining with TiY (10 nM) for 30-40 min. (**C**) Immunofluorescence staining of TiY$^+$ and TiY$^-$ cells sorted from PDX A139 cells for vimentin (green). (**D** and **E**) Tumor sphere forming assay of TiY$^+$ and TiY$^-$ cells. To compare with CD166 on TIC-selective staining of TiY, CD166$^+$ and CD166$^-$ were sorted from the PDX A139 cells when performed

TiY-dependent sorting. Each population (TiY^{+}, TiY^{-}, CD166^{+}, and CD166^{-}) was sorted (5000 cells/ group) and cultured to measure tumor sphere-forming ability. The number of spheres was counted on day 6, and the sphere-forming ability was compared to the total unsorted population (con). Values are means ± SEM (n=3). (**F**) Comparison of tumorigenicity between TiY^{+} and TiY^{-} cell populations of PDX139 cells in mice recipients. (**G**) Immunofluorescence observation of vimentin expression in the paraffin sections of tumor tissues harvested from (F). Scale bar: 50 μm (C), 200 μm (D), 100 μm (G).

Supplementary Figure 5.

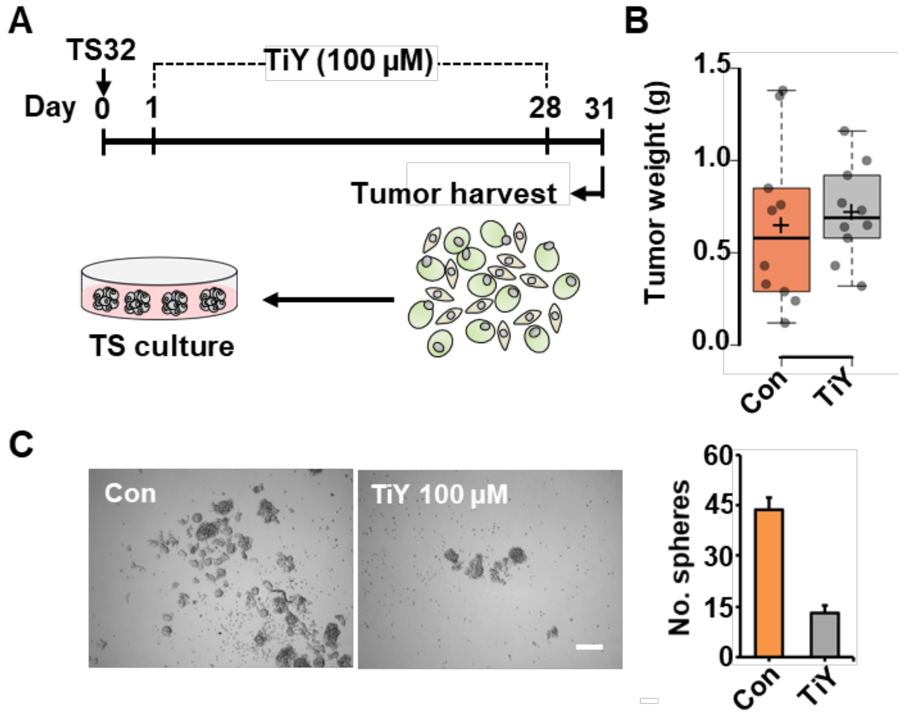

Figure S5. Preliminary study of TIC targeting effect of TiY in TIC line derived xenograft mice

(**A**) A schematic diagram of the experimental procedure for validating TiY's therapeutic effect against TICs in xenograft tumor mice model. One day after the subcutaneous transplantation of TS32 cells into the mice flank, 100 μM of TiY was administrated by the tail vein injection every three days with a volume of 10 μL/ gram until day 28. The tumor size was recorded until day 31, and the tumors were harvested on the day to weigh. The same volume of the vehicle solution was given to the control group mice without TiY (Con). (**B**) The tumor weight of Con- and TiY treated groups. (**C**) Comparing the sphere-forming ability between the Con- and TiY-treated group tumors. Scale bar, 250 μm.

Supplementary Figure 6.

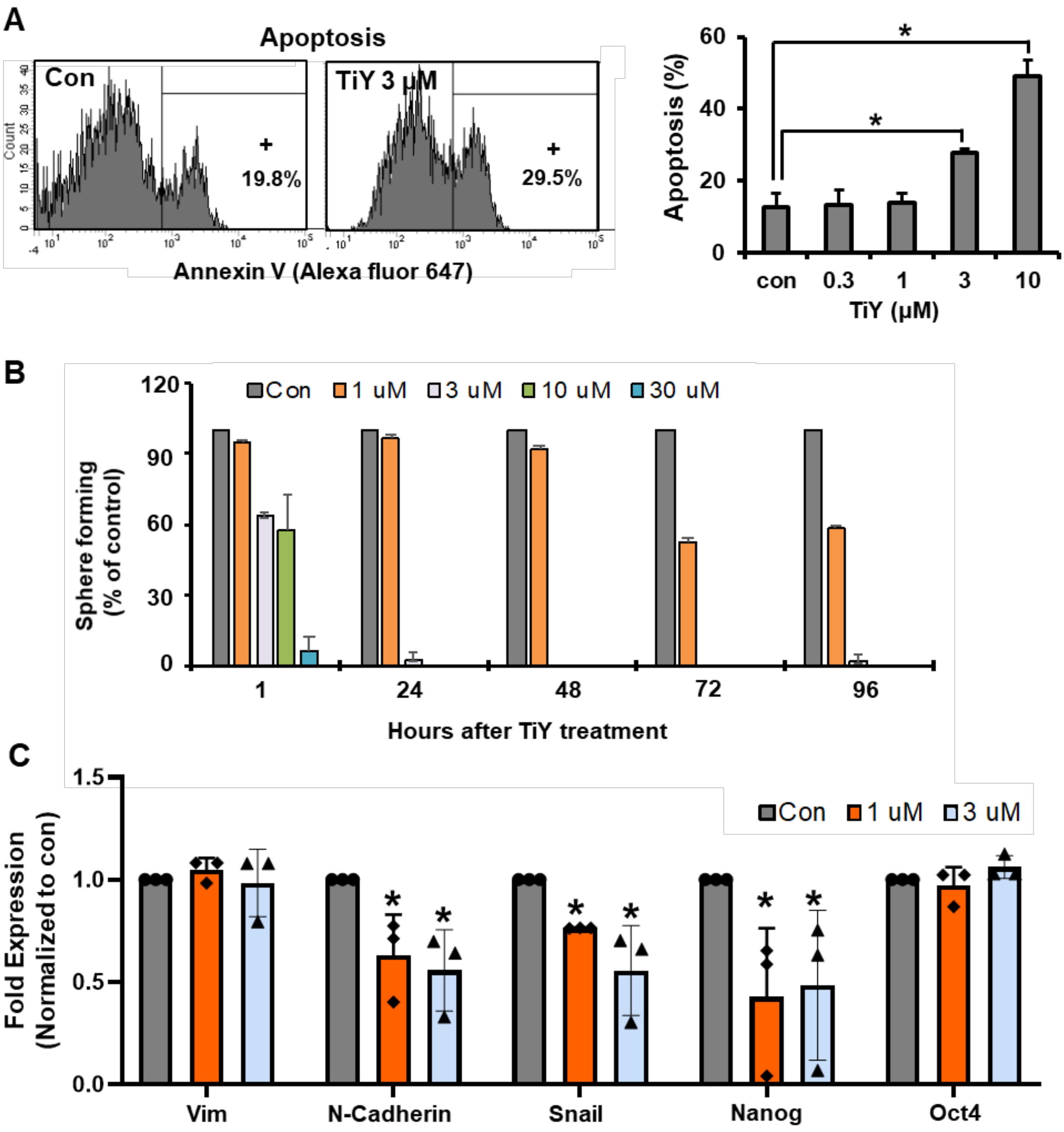

Figure S6. TIC inhibition effect of TiY in TS32 cell culture with different concentrations and treatment hours

(**A**) Apoptotic cell analysis by staining of Annexin V. TS32 cells were treated with TiY with different concentration (0.3, 1, 3, and 10 μM) for 24 hours and harvested to stain with Annexin V-Alexa fluor 647. (**B**) TS32 cells were treated with different concentrations of TiY (1, 3, 10, and 30 μM) for different

time lengths in sphere-forming culture. Data showed that the cytotoxicity was influenced by the increased concentration and treated duration. Values are means ± SEM (n=2). (**C**) qRT-PCR result of TS32 cells after TiY treatment with different concentration (1 and 3 μM) for 24 hours. Values are means ± SE (n=3).

Supplementary Figure 7.

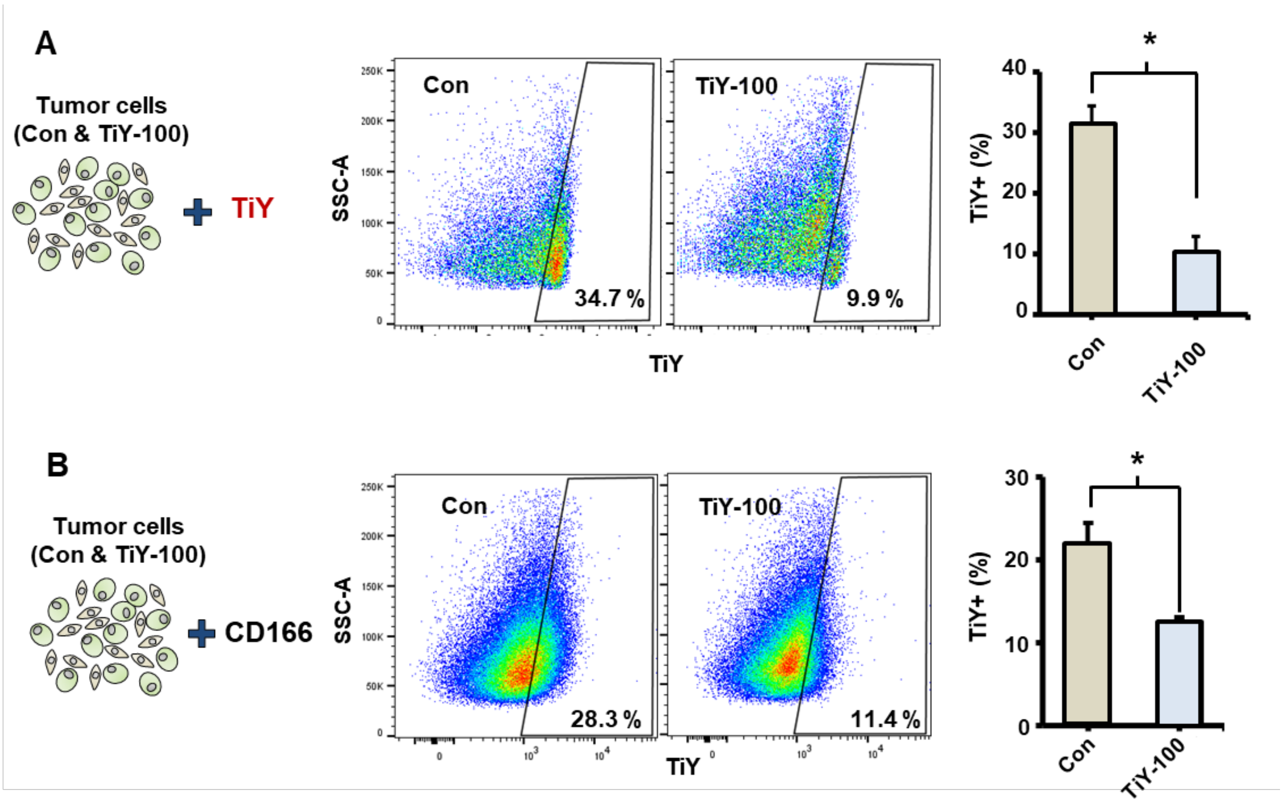

Figure S7. Flow cytometry analysis of tumor cells harvested from control and TiY-100 group

The tumor cells harvested from the control (Con) and 100 μM TiY treated group (TiY-100) mice were subjected to flow cytometry analysis after incubation with TiY (**A**) and CD166 (**B**), respectively. Values are means ± SEM (n=10).

Supplementary Figure 8.

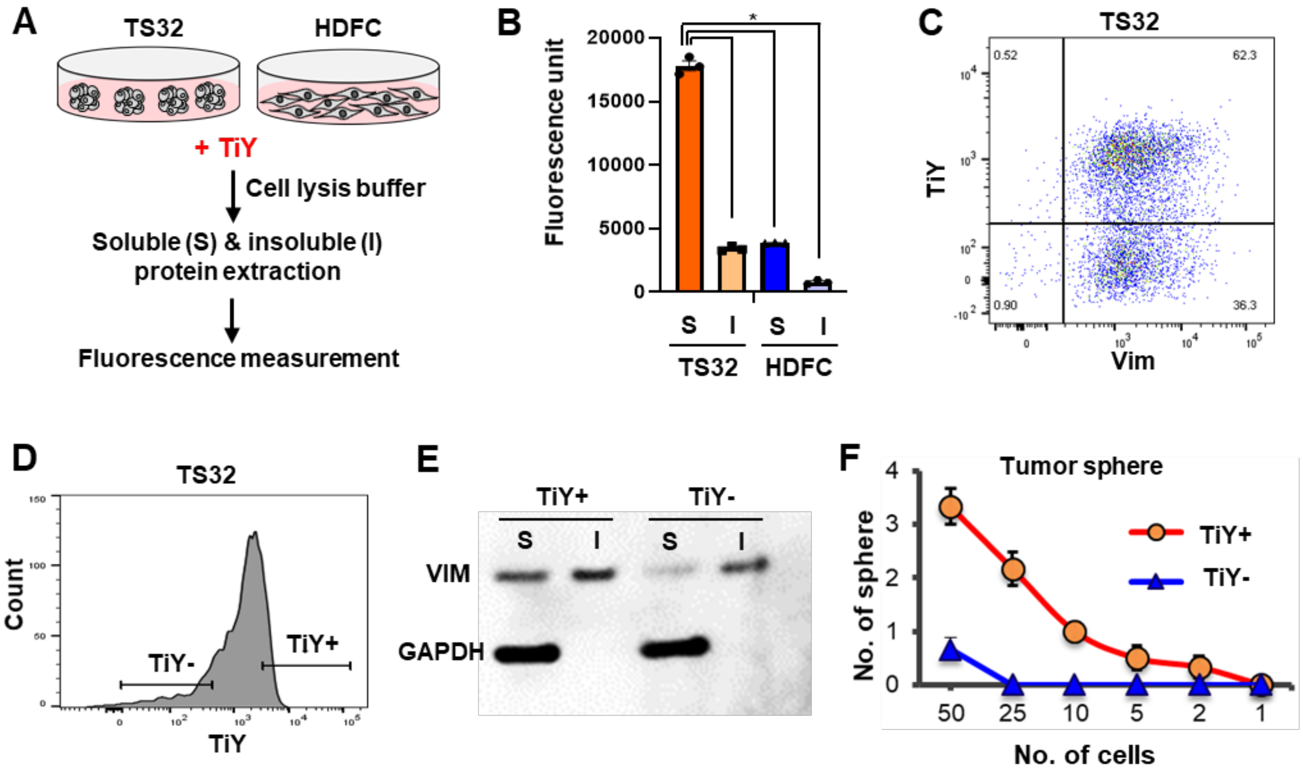

Figure S8. The selective binding of TiY onto the soluble vimentin.

(**A**) Schematic process of validation of soluble vimentin-selective binding of TiY over insoluble counterpart. To validate the TiY affinity onto soluble vimentin, TS32 cells and HDFC were stained with TiY and subsequently extracted for soluble and insoluble protein lysates from the TiY stained cells. (**B**) Comparison of TiY intensity in the soluble (S) and insoluble (I) lysates of TS32 and the human dermal fibroblast cells (HDFC). (**C**) Dual staining of TiY and vimentin antibody in TS32 cells showing an existing a dimly stained population of TiY (TiY$^-$) in vimentin$^+$ TS32 cells. (**D-F**) TiY-dependent cell sorting of the live TS32 cells after TiY staining showed an increased soluble vimentin content and tumor sphere-forming ability in TiY$^+$ cells compared to TiY$^-$ cells.
